# Editorial: Cognitive mechanisms underpinning pro-social behavior across cultures

**DOI:** 10.3389/fpsyg.2026.1820412

**Published:** 2026-04-10

**Authors:** Shijun Xiao, Zijun Huang, Wei Fan, Jin Li, Fangbing Qu, Li Kaiyun, Yiping Zhong

**Affiliations:** 1 Department of Psychology, School of Education Science, Hunan Normal University, Changsha, China; 2 Cognition and Human Behavior Key Laboratory of Hunan Province, Hunan Normal University, Changsha, China; 3 Institute of Interdisciplinary Studies, Hunan Normal University, Changsha, China; 4 College of Preschool Education, Capital Normal University, Beijing, China; 5 School of Education and Psychology, University of Jinan, Jinan, China

**Keywords:** pro-social behavior, cognitive mechanism, cross-cultural, socialization strategies, cognitive development

## 1 Introduction

Pro-social behavior refers to actions intended to enhance the wellbeing of others, encompassing various forms such as cooperating with others, sharing resources, and helping others (Eisenberg et al., 2015). It constitutes a cornerstone of social cohesion and functioning in human societies. For instance, whether it manifests as volunteers spontaneously sharing limited supplies during a natural disaster, or simply a pedestrian pausing to guide a lost stranger in a bustling city, these actions—spanning from extraordinary sacrifices to everyday acts of kindness—weave the invisible fabric of mutual trust that sustains our communities. Research on pro-social behavior has gained momentum since the 1970s, and in recent years, scholars have shifted from mere behavioral description toward in-depth exploration of the underlying mechanisms, developmental trajectories, and context-dependent adaptive processes of pro-social behavior (Henrich et al., 2005; Penner et al., 2005). Accordingly, this Research Topic aims to bring together cutting-edge perspectives and, by comparing individual differences across diverse cultural and social contexts, to provide an in-depth analysis of the cognitive foundations and dynamic processes underlying pro-social behavior. Through the interdisciplinary integration of cognitive science and pro-social behavior research, we hope to offer novel theoretical insights into the core psychological mechanisms that drive altruism, empathy, and social cohesion.

Human behavior does not occur in a vacuum; rather, it is deeply rooted in the surrounding socio-cultural environment. Consequently, the definition, motivation, and manifestation of pro-social behavior may vary considerably across cultures (Köster et al., 2015, 2016). For example, in collectivist cultures that emphasize group cohesion and interdependence, members may exhibit a more pronounced “in-group preference” in their pro-social behavior, prioritizing benefits toward close relations such as family members and friends. By contrast, in individualist cultures that value personal autonomy and universalistic principles (e.g., fairness and justice), pro-social behavior may extend beyond group boundaries to encompass out-group members, including strangers (Kemmelmeier et al., 2006; Triandis, 1989). Moreover, large-scale cross-cultural comparative studies have indicated that cross-societal differences in norms of fairness, punishment, and reciprocity may further shape individuals' behavioral choices in resource allocation and cooperative tasks (Henrich et al., 2010a; Herrmann et al., 2008). Therefore, conducting cross-cultural research to compare both the variability and universality of pro-social behavior across different cultural and social contexts is of critical importance for achieving a more robust understanding of pro-social behavior.

**Figure 1:**
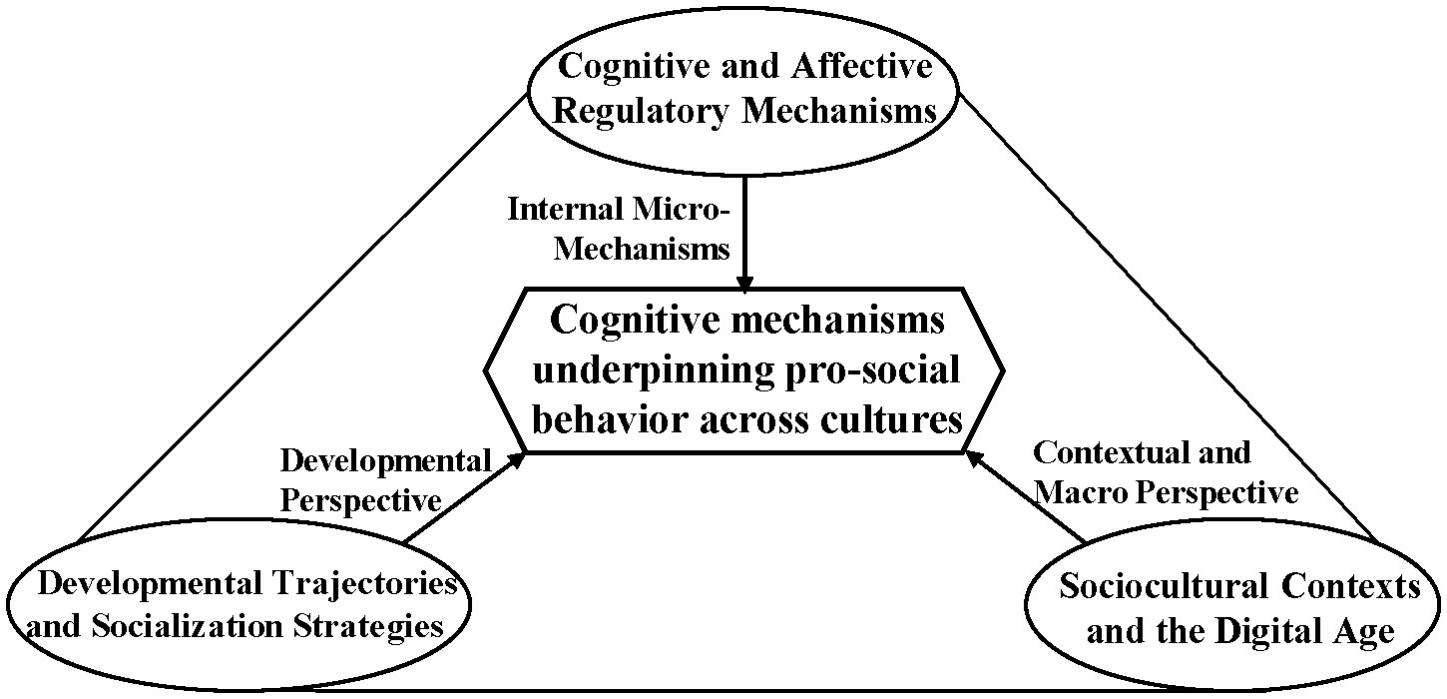
Conceptual framework for the present Research Topic.

This Research Topic ultimately includes 18 scholarly articles. These studies, drawing from a diverse range of perspectives, collectively constitute the rich content of this Research Topic. To provide a clear and intuitive overview of these interconnected perspectives, we present a conceptual framework in Figure 1. Together, they not only showcase the latest research findings in the field but also offer new directions and insights for future investigation.

## 2 Cognitive and affective regulatory mechanisms of pro-social behavior

Far from being a simple, automatic response, pro-social behavior is better understood as an effortful cognitive process that involves actively overriding prepotent selfish impulses and drawing upon finite cognitive resources (Declerck et al., 2013; Rand and Nowak, 2013). The studies in this Research Topic lend strong support to this perspective. Empirical research by Zha et al. highlights the critical dependence of helping behavior on the availability of cognitive resources. Specifically, under conditions of time pressure or ego depletion, individuals struggle to mobilize sufficient cognitive resources to accurately identify others' needs and initiate appropriate helping responses (Diamond, 2013). This resource deficit not only hinders the effective translation of internal helping intentions into concrete pro-social actions (Carlson and Moses, 2001) but also ultimately suppresses the individual's overall pro-social tendencies. However, such resource constraints are not irreconcilable, as emotion regulation capacity serves a buffering role. Gu et al. found that cognitive flexibility plays a critical mediating role between emotion regulation strategies and negative affect. Building on the Conservation of Resources (COR) theory (Hobfoll, 2011; Hobfoll et al., 2018), Liu, Fan et al. constructed and validated a moderated mediation model. Their findings revealed that belief in a just world can function as a core psychological resource that promotes pro-social behavior by enhancing an individual's psychological resilience. Crucially, this mediating process is moderated by empathic ability, manifesting specifically as a “resource compensation effect.” For individuals with low empathic ability, the indirect effect of belief in a just world on pro-social behavior (via psychological resilience) was most pronounced. For their high-empathy counterparts, however, this effect was significantly weaker, suggesting that the cognitive resource of a just-world belief is particularly vital when the emotional impetus for pro-sociality is lacking. Collectively, these findings suggest that pro-sociality is not only influenced by momentary cognitive load but may also be closely linked to individuals' affective states (Gross, 1998).

At a more fine-grained level of information processing, cognitive styles and reward processing mechanisms underpin our social decision-making. Yin et al. found that field-independent vs. field-dependent cognitive styles influence the allocation of attentional resources during individual decision-making, with field-dependent individuals exhibiting heightened sensitivity to and reliance on environmental cues. Furthermore, the internal structure of self-concept plays a critical regulatory role in attentional allocation. Su et al. found that a high level of self-discrepancy induces an attentional bias toward negative information; this bias tends to increase anxiety loads, which in turn can interfere with objective pro-social judgments (Higgins, 1987). Finally, regarding the integration of feedback, Liu, Ding et al. demonstrated that receiving negative external feedback—whether ambiguous or explicit—triggers an effective cognitive calibration, thereby reducing unrealistic positive beliefs during social interactions. In summary, from the allocation of attention to the integration of feedback, these micro-level cognitive processes collectively dictate how individuals interpret complex social contexts and profoundly shape their ultimate social decisions and pro-social behavioral tendencies.

## 3 Developmental trajectories and socialization strategies of pro-sociality

Individuals' pro-social cognition is not static. Many scholars conceptualize pro-social behavior as an early-emerging capacity that is closely intertwined with the development of self-concept, affective resonance, and social cognition (Eisenberg et al., 2015; Imuta et al., 2016). It becomes increasingly complex with age, and numerous studies in this Research Topic have explored this process in depth, delineating how children transition from intuitive helpers to mature agents capable of weighing complex social cues. The study by Yang, Wang et al. showed that children aged 4–6 years are already capable of relatively sophisticated trade-offs between behavioral intentions and outcomes, and can make forgiveness-related choices accordingly. This capacity exhibits consistency across children from different cultural backgrounds (McElroy et al., 2023; Waddington et al., 2023). Following the progressive logic of information processing, Chen, Zha et al.'s investigation of Chinese preschoolers further revealed that 5- to 6-year-olds have developed an emerging capacity for multidimensional information integration. Specifically, when making pro-social decisions, they are capable of simultaneously incorporating multiple factors—such as the fairness of the advice and the familiarity of the advisor—into their evaluative framework. Naturally, these advanced social decisions do not emerge in isolation; the study by Zhen and Liming further demonstrated that the developmental levels of Theory of Mind and empathic ability constitute the core psychological scaffolding that enables children to understand others' needs and generate pro-social responses.

Furthermore, the development of an individual's pro-social behavior is not solely contingent upon the maturation of moral cognitive mechanisms, but is also inevitably shaped by external developmental environments and acquired interventions. Utilizing network analysis, Han and Yan demonstrated that parenting styles, acting as crucial proximal environmental factors, are closely associated with the early development of cognitive and social competencies in preschoolers. At the intervention level, a series of studies by Cai et al., Deng and Xu, and Yang, Peng et al. revealed the positive spillover effects of specific cognitive training regimens. Practices such as music training and mindfulness not only effectively enhance individuals' executive functions but also foster cooperative behaviors through formats like group interactions. Notably, through a three-level meta-analysis, Cai et al. highlighted that the positive impact of music training on children's executive functions is more pronounced in collectivist countries compared to individualist ones. This cross-cultural variance can be attributed to the emphasis that collectivist societies place on group goals, interpersonal cooperation, and social relationships (Chailitilerd, 2014). Importantly, this extension from fundamental complex cognitive training to pro-social actions confirms the presence of significant far-transfer effects. Taken together, these findings not only corroborate the high plasticity of pro-social behavior during early development but also provide a solid empirical foundation for future educational practices and scientific interventions. Future research could consider further exploring how macro-cultural backgrounds and micro-environmental factors (e.g., family environments) interact with specific intervention modalities, and it is recommended to employ longitudinal designs to track the long-term stability of these far-transfer effects.

## 4 Socio-cultural contexts and digital frontiers of pro-social behavior

The final section of this Research Topic broadens the analytical scope to encompass broader socio-cultural and digital contexts, aiming to examine how individuals' pro-social interactions and their underlying psychological mechanisms emerge and evolve within specific external environments. At the micro-level of group interaction, Chen, Zhong et al. found that when individuals collaborate with others, they can construct shared memories, thereby effectively mitigating the continued influence effect of misinformation (Blumen et al., 2014). Such cognitive-level cooperation not only helps defend against misleading content within complex information environments but also lays a solid foundation for future high-quality communication and collaboration among groups. At the macro-level of social ecology, Sun and Yin anchored their research in rural China, uncovering the intricate relationship between the developmental trajectory of individuals' non-cognitive skills and shifts in their socioeconomic status. They found that rural households with stronger non-cognitive skills, such as social initiative and empathy, put their pro-social ideals into practice by forming cooperatives or e-commerce alliances. These cooperative actions not only fostered community cohesion but also brought tangible economic results—enhancing collective wellbeing and effectively alleviating relative poverty. The impact of these non-cognitive skills on poverty reduction for rural households is not static; it is significantly moderated by the macro-environment. In the economically less developed western regions of China, the poverty-alleviating effect of improved non-cognitive skills is far greater than in the eastern and central regions. This research not only provides valuable localized empirical evidence but also reaffirms the profound shaping effect of macro-environmental systems on individual psychological and behavioral development (Conger and Donnellan, 2007).

With the advancement of digital technologies, human interaction is no longer confined to face-to-face communication, offering novel perspectives for research on pro-social behavior. You et al. found that in digital interactions, when participants communicated with users bearing feminine-sounding usernames, they perceived greater friendliness and social attractiveness, which in turn increased their willingness to engage in pro-social behavior. Going further, Wang et al. found that engaging in virtual pro-environmental behavior in metaverse spaces may influence individuals' pro-environmental behavior in the real world. Particularly after being assigned an eco-friendly label, individuals' environmental self-identity is reinforced, thereby motivating more authentic pro-environmental actions. This resonates with the Proteus Effect proposed by Yee and Bailenson (2007), which posits that the characteristics of virtual avatars can reciprocally reshape individuals' behavioral patterns. These findings provide a novel and solid empirical reference for understanding how environmental cues—whether real or virtual—modulate human cooperation and altruistic behavior.

## 5 Conclusion

Overall, the series of studies compiled in this Research Topic, anchored in a cognitive perspective, systematically presents key findings and cutting-edge insights into the psychological mechanisms underlying cross-cultural pro-social behavior. Within the dimension of cognitive and affective regulation, the relevant research not only precisely identifies the core executive functions that underpin pro-sociality but also uniquely reveals how cognitive resource states (e.g., ego depletion) and emotion regulation strategies work in tandem to shape individuals' social decision-making processes. Regarding developmental trajectories and intervention practices, this issue delineates effective pathways for cultivating pro-social traits—ranging from early proximal parenting environments to acquired music and mindfulness training. These findings are of paramount importance for profoundly understanding the high plasticity of individuals' social-cognitive abilities and their dynamic evolutionary mechanisms. Finally, in terms of socio-cultural and digital contexts, the scope of research extends seamlessly from physical reality (such as macro-level socioeconomic status) to digital spaces (such as virtual interactions in the metaverse). These articles comprehensively analyze how multidimensional environmental cues cross-contextually modulate human cooperation and altruism, further corroborating the sustained spillover effects elicited by these external factors.

Notably, the majority of studies included in this Research Topic originate from non-Western cultural contexts, which not only broadens the literature base that has long been dominated by Western samples (Henrich et al., 2010b) but also tests and refines the cross-cultural applicability and contextual contingency of existing pro-social behavior mechanisms. For example, classical theories of moral development often suggest that as children age, their moral judgments gradually shift from being “outcome-oriented” to “intention-oriented.” A study by Yang, Wang et al. on in-group and out-group interactions among Chinese children found that children flexibly weigh intentions and outcomes when making judgments, and this weighing strategy changes depending on whether the other party is an in-group or out-group member. This fully demonstrates the cross-cultural applicability of this pro-social decision-making mechanism. Furthermore, a meta-analysis by Cai et al. discovered that the enhancing effect of music training on children's executive functions (a foundation for pro-social behavior) is more significant in collectivistic cultural contexts than in individualistic ones. This clearly indicates that even seemingly universal intervention methods can have effects that vary with cultural context, and therefore need to be tested and optimized through cross-cultural comparison and localized practice. All these studies remind us that we need to remain sufficiently sensitive to cultural variables when constructing a general theory of pro-social behavior. We hope that by bringing together empirical evidence from diverse cultural backgrounds, this Research Topic can offer a meaningful contribution to understanding the cognitive roots of human pro-social behavior and provide a reference for future systematic research conducted across broader cultural contexts and increasingly complex technological environments.
